# Long-Term Survival of Mandibular Incisors with Severe Periodontal Breakdown: Mean Follow-Up of 18 Years

**DOI:** 10.3390/jcm15062129

**Published:** 2026-03-11

**Authors:** Ben De Backer, Hein De Backer, Georges Van Maele, Selena Toma, Véronique Christiaens

**Affiliations:** 1 Department of Periodontology, Cliniques Universitaires Saint Luc, Université Catholique de Louvain (UCL), 1200 Brussels, Belgium; selena.toma@saintluc.uclouvain.be (S.T.); 2 Private Practice of Periodontology and Prosthetic Dentistry, 8700 Tielt, Belgium; 3 Faculty of Medicine and Health Sciences, Oral Health Sciences, Department of Gnathology, Temporomandibular Disorders and Oral Facial Pain, Ghent University, 9000 Ghent, Belgium; 4 Biostatistics Unit, Faculty of Medicine and Health Sciences, Ghent University, 9000 Ghent, Belgium; gvnmaele@gmail.com; 5 Faculty of Medicine and Health Sciences, Oral Health Sciences, Department of Periodontology and Oral Implantology, Ghent University, 9000 Ghent, Belgium; vchristi.christiaens@ugent.be

**Keywords:** periodontitis, severe alveolar bone loss, subgingival debridement, tooth splinting, mandibular incisors, long term survival

## Abstract

**Background:** This retrospective study assessed long-term survival outcomes of severely periodontally compromised mandibular incisors (≥50% bone loss) following initial periodontal treatment and a structured recall protocol. **Methods:** Ninety-three patients with ≥50% bone loss in all mandibular incisors were treated in a private practice over a 32-year period by the same periodontist. Following initial treatment, patients were assigned 6- or 12-month recall intervals based on response and motivation. The baseline was set after subgingival debridement (visit 3). Last follow-up visit (LFV) in this study was defined as follows: the last control visit of the patients done by the periodontist. ‘Survival’ was divided into 3 groups: complete survival (CS), all incisors were still present, and partial survival (PS), one or two incisor(s) were lost. Total failure (TF) involved instances in which all incisors were lost. Effective survival was monitored when an extracted tooth was repositioned and stabilized with a splint, ensuring preservation of function. Only 9.7% of patients needed a mandibular incisal splint. For reasons of consistency the CPITN was used. Statistical analysis was performed in R. The significance level was set at α = 0.05. Event-free patients can be considered as uninformative censoring, all with the same probability of risk, as they all were still in follow-up at the time of informed consent approval. **Results:** A total of 93 patients were included in the study. The mean follow-up was 17.7 years. At the last visit, 79.6% of patients retained all incisors, with an effective survival rate of 89.2%. Regarding the survival probability over time, after 15 years, it is 91% (95% CI: 0.86–0.98), and after 20 years, it is 78% (95% CI: 0.69–0.90). The effective survival probability over time after 15 years was 95% (95% CI: 0.91–1.0), and after 20 years, it was 89% (95% CI: 0.81–0.98). Compliance significantly influenced survival (*p* = 0.007), whereas the number of occluding units did not (*p* = 0.226). The total amount of teeth lost during the entire follow-up period showed a statistically significant difference compared to survival (*p* < 0.001). The general periodontal health of the patient population presented a shift from CPITN 3 to the 0–2 group. **Conclusions:** Severely compromised mandibular incisors demonstrate high long-term survival rates with appropriate therapy. After 20 years the survival probability was 78%, and the effective survival probability, 89%, underscoring the critical role of lifelong periodontal care. Mandibular incisor preservation over long-term follow-up is highly achievable.

## 1. Introduction

Although periodontitis is a multi-factorial disease, the susceptibility and progression of this disease is largely determined by the host inflammatory response [1,2]. In addition to environmental and behavioural risk factors, genetic variations may influence this host response. Interleukine-1 (IL-1) polymorphisms represent genetic variations in the IL-1 gene cluster and have been associated with increased cytokine production and an exaggerated inflammatory response to bacterial plaque. This may result in accelerated attachment loss and alveolar bone resorption, potentially leading to more severe periodontal breakdown [3]. However, genetic testing is currently not part of standard periodontal diagnostics or treatment protocols in private practices. Severe periodontal breakdown affects quality of life, not only in terms of reduced functional capacity but also social and interpersonal relationships [4]. Papapanou et al. (2018) [5] defined a multi-dimensional staging and grading system for periodontitis, replacing the aggressive and chronic periodontitis classification [6].

The combination of periodontal breakdown and increased mobility frequently leads to an irreversible decision, namely tooth extraction. However, this decision is based on clinical and radiographic parameters, combined with the scientific and personal knowledge of the treating dentist. There may be different interpretations of when to extract a tooth. Critical diagnosis and adequate treatment can lead to a decrease in mobility over time, if not to tooth extraction [7]. The treatment planning of the practitioner is crucial and is often influenced by experience. A tooth that may be considered severely compromised for one dentist could have a poor prognosis according to another one, while a third may consider long-term retention feasible under strict periodontal treatment and maintenance protocols [8]. Therefore, it would be interesting to know what the mean survival time is of teeth that are classified as questionable or considered severely compromised based on their periodontal condition [9].

Especially with the increasing average life expectancy of the global population [10] a back-off strategy is proposed in several instances [11] for people with complicated and/or full-mouth (fixed) rehabilitations. Often all four incisors are extracted, although only one or two have lost ‘excessive’ bone, as replacing one or two mandibular incisors appears to be an (aesthetically) inferior option. Consequently, the remaining incisors might be sacrificed, but this could induce an irreversible change in the oral situation of these patients. Instead of extracting all incisors, an option is to splint the extracted tooth to its neighbouring teeth. Although this treatment option was documented before [12,13], the overall lifespan of this temporary treatment is not well known.

This retrospective study investigates the average survival rate as well as ‘effective survival’ of mandibular incisors with a minimum of 50% supportive tissue loss. In addition, the effect of compliance, occluding units and CPITN in relation to the general periodontal condition of the patients was assessed on their survival.

## 2. Materials and Methods

All patients included were treated by the same periodontist over a period of 27 years (1990 to 2017) in a private practice. The retrospective end-evaluation was set up on 31 October 2022. The maximum follow-up period was 32 years (1990 until 2022). In total 93 patients were included. None of the patients of the drop-out group were contacted by telephone and no questionnaires were sent to them, nor to current clinicians of these patients to collect supplementary information.

‘Survival’ was divided into complete survival (CS), all incisors were still present at last evaluation, and partial survival (PS), one or two incisor(s) were lost during the study. None of the patients lost three incisors. Total failure (TF) involved instances in which all incisors were lost.

‘Effective survival’ was defined as follows: patients experienced the loss of one or two mandibular incisors during the study follow-up. The extracted teeth were repositioned and stabilized with a splint, ensuring preservation of function. This subgroup could be considered as part of the overall survival cohort, as the functional and aesthetic integrity of all mandibular incisors were maintained.

Acknowledgement from the Ethical Committee at the University of Ghent was obtained with reference number ONZ-2022-0608. An Informed Consent Form (ICF) of the Ethical Committee was used. After complete explanation of the study, all patients included signed the ICF. This study was conducted in accordance with the principles of the Declaration of Helsinki.

All patients were selected based on clear criteria:

Inclusion criteria

- Periodontitis class 3C or 4C, according to Papapanou et al. (2018) [5].
- All mandibular incisors had <50% supporting bone.
- All ASA scores.

Exclusion criteria

- Insufficient radiographic examinations available.
- Absence of periodontal information.
- Follow-up time < 5 years.

### 2.1. Patients’ Care Pathway and Evaluation of Patients’ Files

Data were extracted from the patients’ files: initial periodontal assessment (visit 1) and initial periodontal treatment (IPT), including subgingival debridement (SGD) and extractions if indicated (visit 2 and 3 = baseline). After SGD patients had three more follow-up visits (4 to 6): at 1, 3 and 6 months. At visit 6 a full periodontal status was taken (general re-evaluation, GR) and compared to the initial status. All visits were performed by the same periodontist. In Belgium, the assistance of dental hygienists was legally not possible until 2019. Additional oral hygiene instructions were provided. Antibiotics were never prescribed, neither before, during nor after periodontal treatment. The periodontist maintained the same treatment and maintenance protocol over a 32-year period. During maintenance a new periodontal status was taken at each visit, as well as full-mouth supra-gingival debridement, together with a motivation of personal plaque control. Sub-gingival debridement was performed when necessarily. Patients were informed that the periodontal condition of their mandibular incisors was extremely doubtful. If mandatory, a splint was placed prior to IPT.

After IPT, a recall period of 6 or 12 months was appointed based on patient’s response to treatment and motivation. Last follow-up visit (LFV) in this study was defined as the last control visit of the patients done by the periodontist before 31 October 2022. Patients were not invited for an additional visit in the context of this study. The follow-up frequency (FUF) was created to indicate the difference between individuals adhering to strict periodontal recall visits and individuals with irregular maintenance visits.

Based on patient records, compliance was assessed retrospectively using predefined criteria primarily related to attendance at supportive periodontal therapy visits. Patients were initially categorized into five compliance levels (‘excellent’, ‘good’, ‘moderate’, ‘bad’ and ‘no compliance’) based on follow-up frequency and appointment adherence. For analytical purposes, these categories were dichotomized into ‘compliance’ (‘excellent’, ‘good’, and ‘moderate’) and ‘non-compliance’ (‘bad’ and ‘no compliance’). Patients classified as compliant attended regular follow-up visits at intervals of six or twelve months, whereas non-compliant patients showed irregular attendance or long interruptions in follow-up. Periodontal examination results were used descriptively to characterise the groups but were not used as primary criteria for compliance classification. Compliance classification was performed by a single experienced periodontist using standardized criteria to ensure consistency. Although some degree of subjectivity is inherent to retrospective file-based assessments, the use of predefined attendance-related criteria aimed at minimizing misclassification and observer bias.

### 2.2. Periodontal Index

In this retrospective study data from the initial and last comprehensive full-mouth periodontal screening was used. The Dutch Periodontal Screening Index (DPSI) [14] was used as the primary tool for periodontal assessment, derived from the Community Periodontal Index of Treatment Needs (CPITN) [15]. For the interpretation of the results, DPSI scores were seamlessly converted to their corresponding CPITN values.

### 2.3. Radiographic Evaluation and Measuring Protocol

To assess the bone loss on all mandibular incisors, the full root length and the most severe site of bone resorption on each of the incisors were examined on analogue radiographs (X-ray visit 1). The relative bone loss per tooth was calculated as follows: divide the total root length by the length of the root still covered in the mandibula at the most impacted location of the tooth × 100.

Different instruments were examined: a standard periodontal probe (accuracy up to 1 mm), a calliper (CA) (up to 0.1 mm) and an electronic calliper (up to 0.01 mm). Based on the intraclass correlation, the reliability of the researcher between the two assessments and between the two consecutive days were good to excellent (Table 1). To assess procedural accuracy and researcher reliability (BDB), root length and bone resorption of the most affected tooth were measured using three instruments in 10 randomly selected cases. This procedure was repeated after one hour. The researcher measured the same teeth twice more with a one-hour brake on a different day. All measurements were accurate or excellent with all three different tools, meaning that the measuring accuracy by our one investigator suffices. A Bland–Altman test revealed that the most accurate measurements on an analogue radiograph were made by using a CA. Therefore, the CA was used for all the measurements.

### 2.4. Statistical Analysis

Statistical analysis was performed in R [16,17]. A univariate comparison of unpaired groups was done with Fisher’s Exact test for categorical data. The Mann–Whitney U-test, Wilcoxon-matched-pairs signed-ranks test and Kruskal–Wallis ANOVA were used to compare ordinal and continuous variables. The preference for non-parametric statistics is justified in the context of often skewed data and small sample sizes.

Survival rates were calculated by the method of Kaplan and Meier [18], with a 95% confidence interval. Event-free patients can be considered as uninformative censoring, all with the same probability of risk, as they all were still in follow-up at the time of informed consent approval. No further sensitivity analysis for overall survival has been performed. The log-rank test was used to discover whether some survival functions differ between groups, whereby the effect of co-variables was analysed by Cox regression [19]. The significance level was set at α = 0.05. The patient was set as statistical unit.

## 3. Results

All patient records from a private periodontal practice were screened to identify patients diagnosed with stage III/C or IV/C periodontitis. Additional exclusions were applied when not all four mandibular incisors demonstrated at least 50% bone loss. Complete treatment and follow-up records were initially available for 100 patients; however, seven were subsequently excluded due to missing periodontal parameters or radiographic data. However, these drop-outs did not show any complications at LV. Consequently, 93 patients were included in the final study population.

The mean follow-up period was 17.7 years (5–32 yrs). The gender distribution was homologous (males: 46.2%, females: 53.8%). The mean age was 49.7 years (33–73 yrs), with no statistically significant (SS) difference in survival (*p* = 0.46). Similar results were found for the gender distribution (*p* = 0.37). Follow-up frequency varies between 5.3 and 31.9 years. After 10 years, 82.8% of patients *(n* = 77) remained in the study, and at 15 years, 65.6% (*n* = 61). By 20 years, 34.4% of patients (*n* = 32) remained in the study; after 25 years, 19.4% (*n* = 18); and after 30 years, 2.2% (*n* = 2). The group consisted of 13 smokers, with no SS difference in survival (*p* = 0.310). Constant findings for survival were observed in diabetes patients (*n* = 6; *p* = 0.380) and those with bisphosphonate use (*n* = 3; *p* = 0.140), as well as for the daily consumption of four or more medications (*n* = 27; *p* = 0.970). None of the patients needed additional surgical treatment. Only re-instrumentation of sites with persisted bleeding on probing (BoP) and probing pocket depths (PPDs) of ≥5 mm were performed.

**Figure 1:**
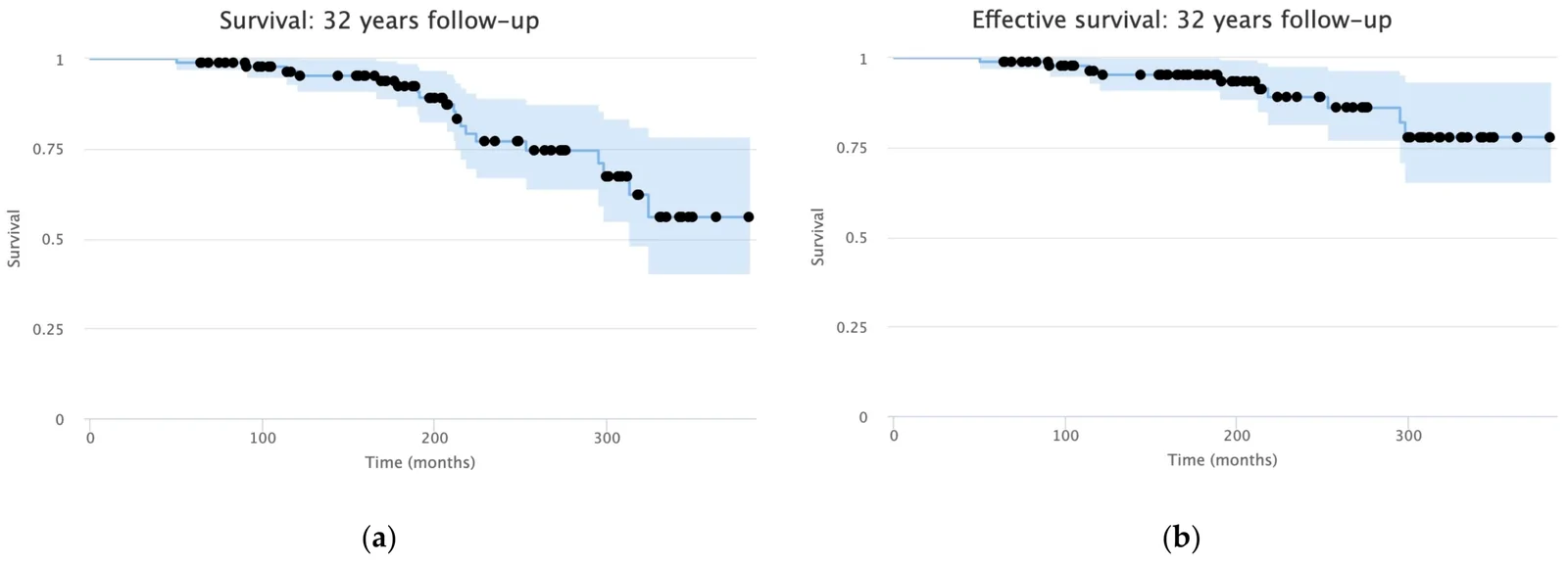
(**a**) Survival probability after 32-year follow-up; (**b**) effective survival probability after 32-year follow-up.

The re-evaluation at LFV showed a survival distribution of 79.6% for CS, 12.9% for PS and 7.5% for TF. In the TF group (*n* = 7), the initial failure occurred after 7.6 years (2nd: 10.1, 3rd–7th: within the range of 17.5–24.8). In the PS group (*n* = 12), none of the patients lost three teeth. Only 9 out of 12 lost one tooth. In this last group, the initial event was 9.5 years into the follow-up, and the last patients, after 27.0 years. Only three patients lost two teeth (range: 17.9–24.6 yrs). Regarding the survival probability (Figure 1), after 5 years of follow-up there is 99% (95% CI: 0.97–1.0) survival. At the 10-year follow-up, the survival probability is 94% (95% CI: 0.89–0.99); 15-year follow-up, 91% (95% CI: 0.86–0.98); and 20-year follow-up, 78% (95% CI: 0.69–0.90). The survival of all mandibular incisors independently indicates that tooth 3.1 was lost 14 times; tooth 4.1, eleven times; tooth 3.2, ten times; and tooth 4.2, only 8 times. The mean survival time up to 25 years is respectively 75%, 77%, 78% and 83% per tooth.

Regarding the general periodontal health of the patient population, at GR 34 patients scored a CPITN of 0–2. Scores of 3 and 4 were documented for 53 and 6 patients respectively. When evaluating this index at LV, there were 42, 42 and 9 patients in the CPITN scores of 0–2, 3 and 4 respectively, showing a shift from CPITN 3 towards the 0–2 group. For the overall number of failures (partial and total, *n* = 19) 5 failed with a general CPITN score of 0–2; 9 failed with a score of 3; and 5 failed with a score of 4.

During the study follow-up, several patients (*n* = 12) experienced the loss of one or two mandibular incisors. In 8 cases, the extracted tooth was repositioned and stabilized with a splint, ensuring preservation of function. One patient lost two incisors, which were similarly managed with this approach. These 9 patients in this subgroup completed the study with their repositioned tooth/teeth. At LFV, this ‘effective survival group’ showed an effective survival distribution for the different groups of 89.2% for CS, 3.2% for PS and 7.5% for TF. Regarding the effective survival probability over time, after five years of follow-up there is 99% (95% CI: 0.97–1.0) effective survival. At the 10-year follow-up there is 95% (95% CI: 0.91–1.0) effective survival; 15-year follow-up, 95% (95% CI: 0.91–1.0); and 20-year follow-up, 89% (95% CI: 0.81–0.98).

**Figure 2:**
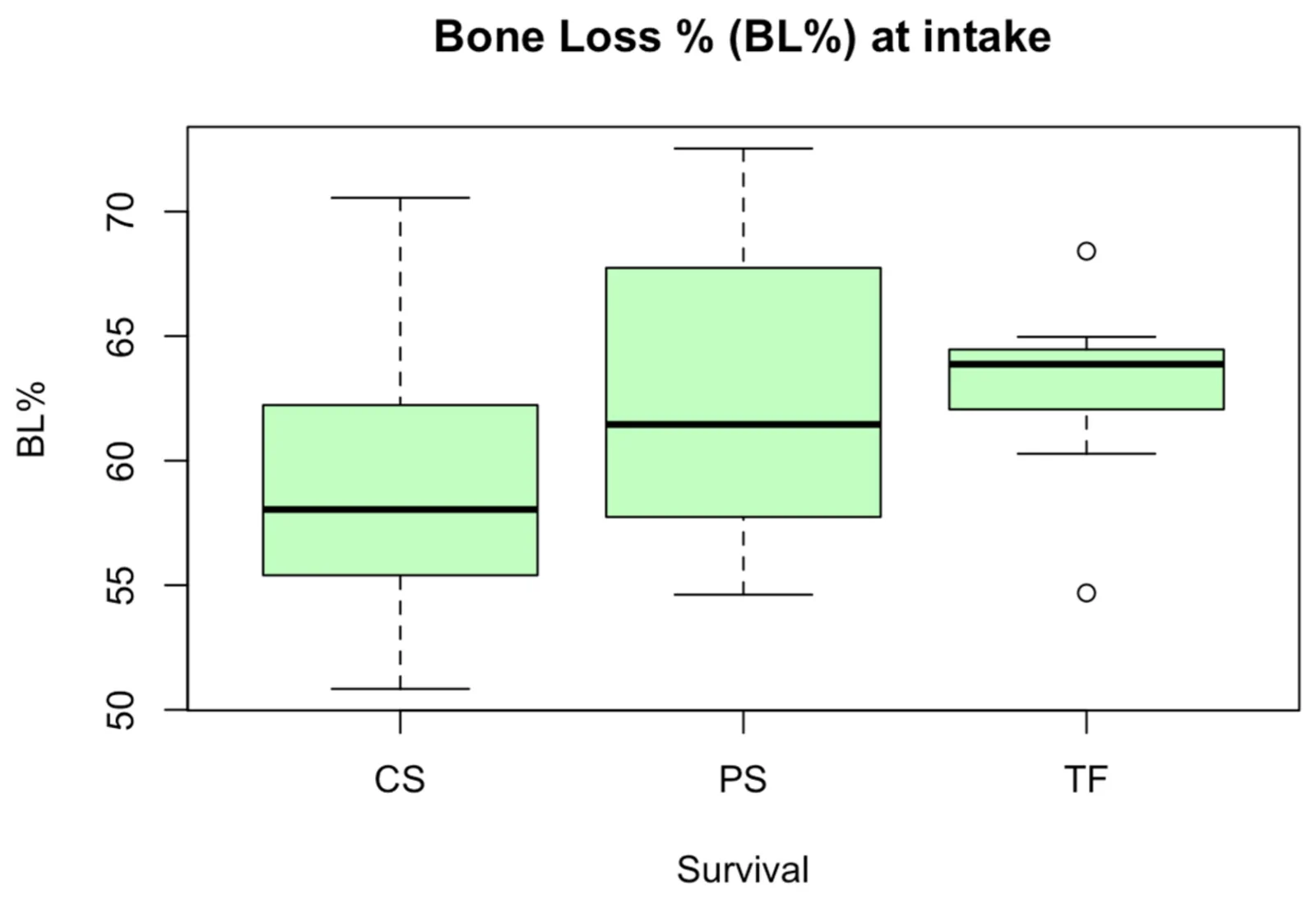
Comparing survival to mean bone loss at intake revealed a statistically significant difference between groups (*p* = 0.21).

Bone loss was measured throughout the different groups and time points: at intake (Figure 2) and LFV. Comparing survival to mean bone loss at intake revealed an SS difference between groups (*p* = 0.021). The mean bone loss was 58.8% in the CS group, 62.5% in the PS group, and 62.9% in the TF group. At LFV, the CS group had a mean bone loss of 60.0%, while the PS group showed 64.9% mean bone loss. The difference was borderline SS (*p* = 0.070) between these two groups.

**Figure 3:**
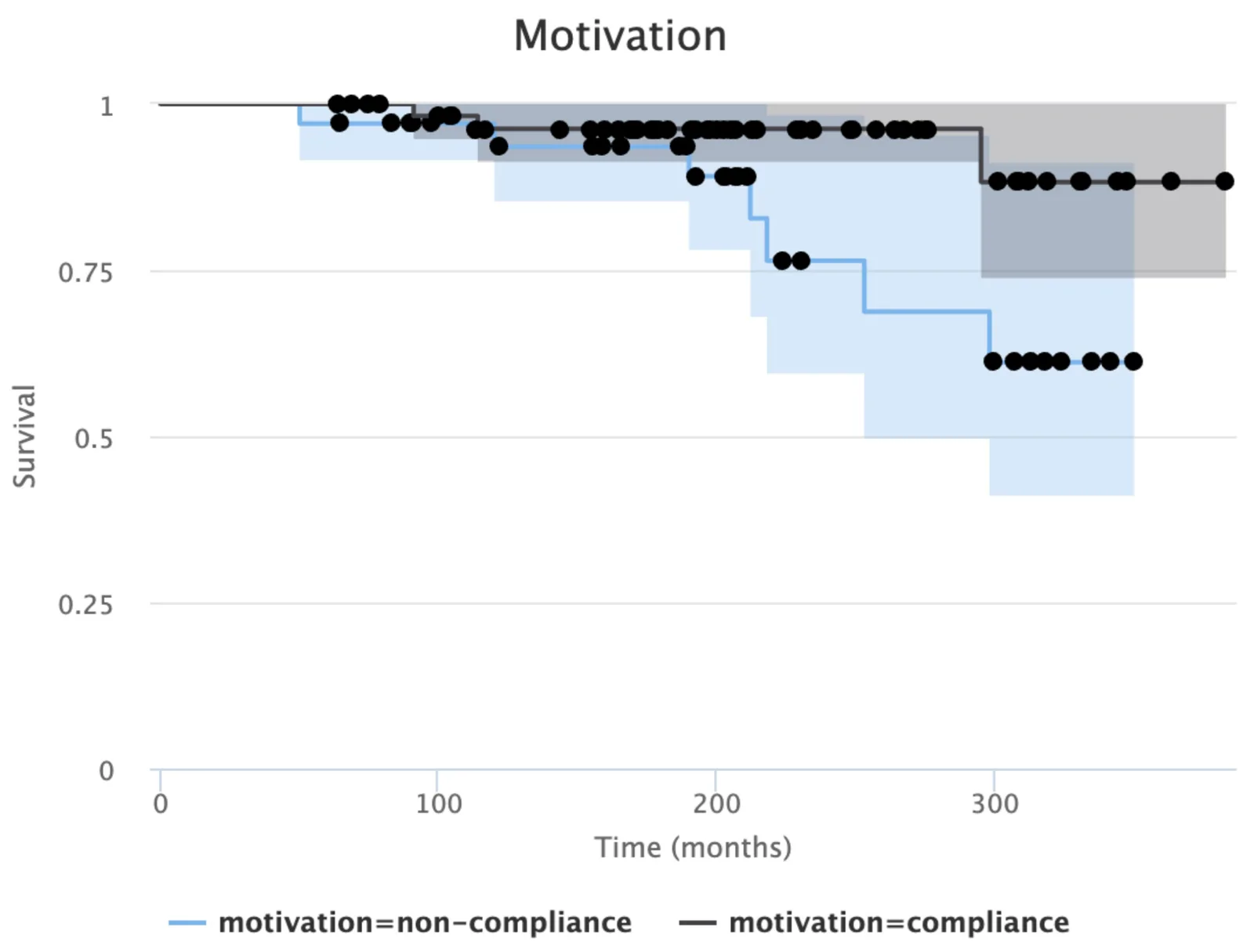
Comparison of motivation to survival. The SS result was confirmed (*p* = 0.007).

The *p*-values for both compliance groups were significant (*p* < 0.001 and *p* = 0.014 respectively) according to Kaplan–Meier (Figure 3). When comparing motivation to survival, the SS result was confirmed (*p* = 0.007). The effective survival rate for the CS group in the non-compliance group is 64.7%, whereas it is 88.1% in the compliance group. Considering the difference in motivation between men (60.5%) and women (66.0%), no SS difference was observed (*p* = 0.667). Cox regression, as a multivariate approach to control for potential confounders, such as age, gender, medication usage, smoking and diabetes, showed no effect on the significant differences in survival outcome.

Treatment effects compared on bone loss percentages at intake and LFV did not SS differ between the CS and PS group (*p* = 0.521). The average bone loss percentage at intake was compared to both compliance groups. For the compliance group, no SS difference for survival was observed (*p* = 0.986). On the contrary for the non-compliance group there was an SS difference (*p* = 0.033).

Splinting before initial therapy was SS associated with mandibular incisor survival (*p* = 0.012). In the patient group (*n* = 10) with splinted teeth, four patients lost teeth (one patient lost one tooth, one lost two teeth, and two lost all incisors). In this group 100% of tooth loss resulted from periodontal disease. The splint cohort accounted for 35.4% of periodontal mandibular incisor losses (*n* = 11 of 31) in the overall study population. In total 43 incisors were lost, and in the non-splinted group, 20 for periodontal reasons. Another 12 were lost due to other reasons.

Patients with a periodontal splint had on average 6.1% greater initial bone loss at intake than those without (65.0% vs. 58.9% respectively, *p* < 0.001). This may contribute to the increased risk of mandibular incisor loss, with an odds ratio of 4 in the splinted cohort. Bone loss at intake was SS greater in patients who lost a mandibular incisor due to periodontal reasons compared to those who did not (mean percentages of 62.6% vs. 59.0% respectively, *p* = 0.020).

Follow-up frequency was categorized into regular and irregular maintenance groups, showing borderline SS (*p* = 0.062), possibly due to a lack of statistical power. The regular maintenance group achieved 82.1% in the CS category, while the irregular group obtained 55.6% in the CS category. Considering the difference in FUF between men (90.7%) and women (90.0%), no SS difference was observed (*p* = 1.0).

**Figure 4:**
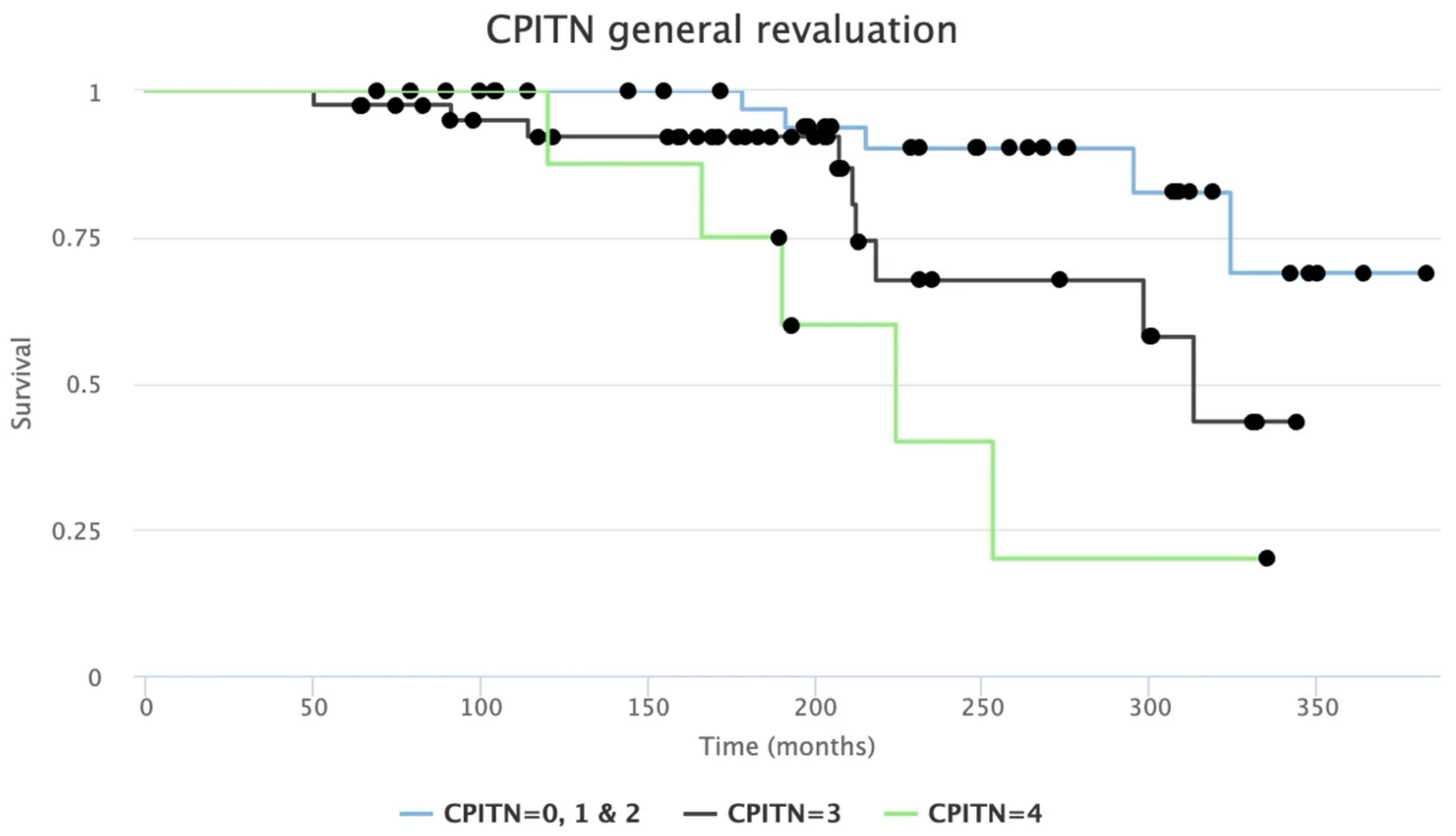
Mandibular incisors showed a statistically significant lower survival probability when CPITN 4 was observed at general re-evaluation (*p* = 0.004).

In relation to the periodontal status, mandibular incisors showed an SS lower survival probability when CPITN 4 was observed at GR (*p* = 0.004) (Figure 4) compared to the groups “CPITN 0–2” and “CPITN 3”. This trend persisted at LFV (*p* = 0.0024).

**Figure 5:**
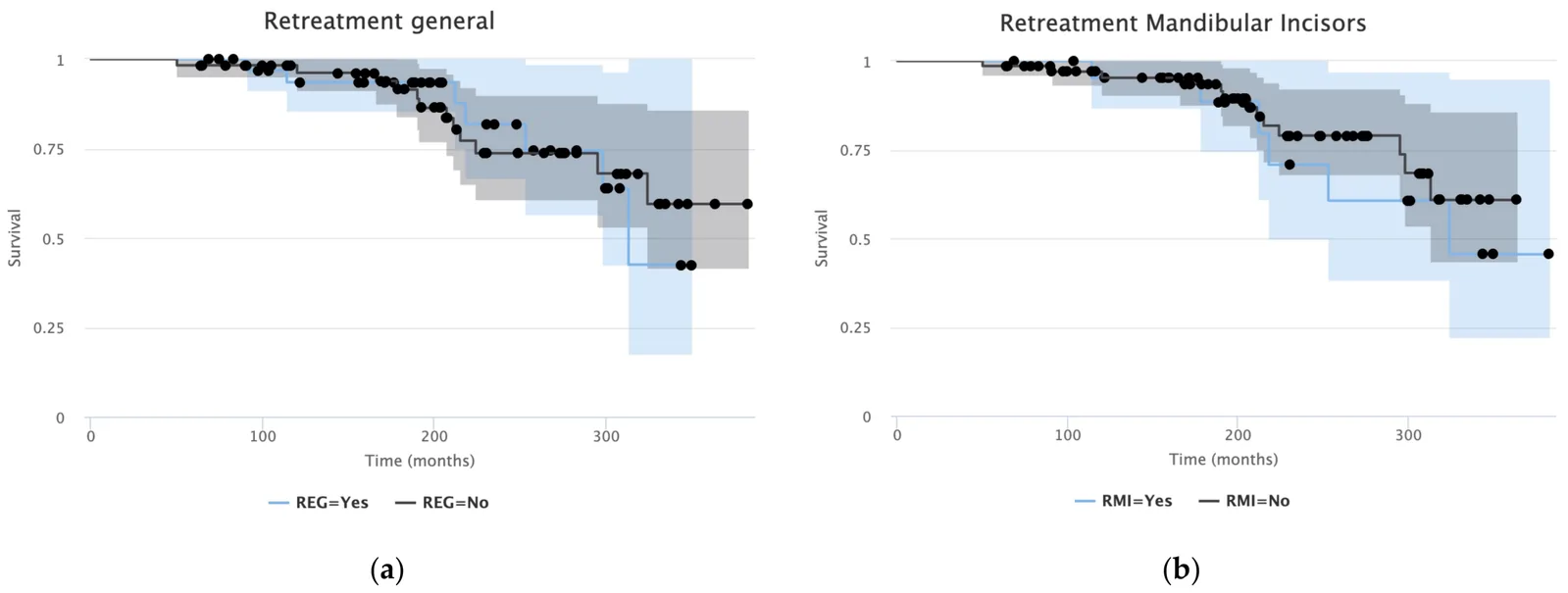
(**a**) Survival probability when a general retreatment was performed; (**b**) survival probability when mandibular incisors were retreated. Retreatment protocol effectively maintained mandibular incisor retention independent of the need of site-specific intervention.

The impact of a full-mouth retreatment throughout the whole follow-up period (*n* = 35) (involving non-surgical intervention pockets ≥ 5 mm) SS demonstrated no additional loss of mandibular incisors compared to patients who did not require retreatment (*p* = 1.0). Among the 35 patients undergoing retreatment, 23 also required retreatment of the mandibular incisors. The statistical analysis revealed no SS difference in tooth loss when compared to no mandibular incisor retreatment (*p* = 0.510). These findings (Figure 5) emphasise the efficacy of the retreatment protocol in maintaining mandibular incisor retention irrespective of the need for site-specific retreatment.

When interpreting the results in the context of overall oral health status, the following findings were observed. Between the three different groups the number of occluding units did not significantly impact survival (*p* = 0.226). Meanwhile, the total amount of teeth lost during the entire follow-up period showed an SS difference compared to survival (*p* < 0.001).

The loss of mandibular incisors (*n* = 43) can be attributed to: periodontal issues (*n* = 31), aesthetic reasons (*n* = 8), medication use (*n* = 1), endodontic reasons (*n* = 2), and root fracture (*n* = 1). The reasons for loss of all incisors at once include: aesthetic reasons (*n* = 2), after 91 and 211 months, and periodontal breakdown: 5 patients (range 122–298 months).

**Figure 6:**
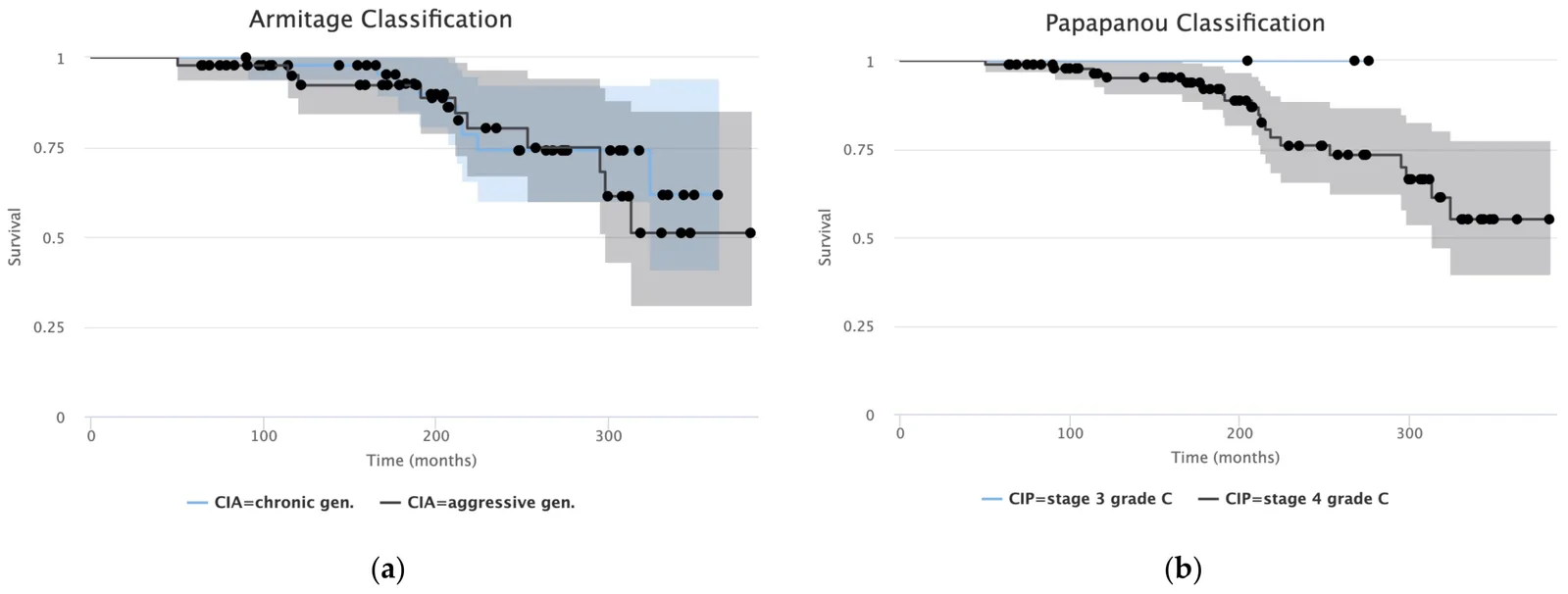
(**a**) No significant difference in survival was observed between chronic and aggressive periodontitis (*p* = 0.60); (**b**) the updated classification demonstrated a greater concordance with the outcomes of the present study.

According to the periodontal classification of Armitage et al., both groups (chronic and aggressive periodontitis) showed no significant differences in survival (*p* = 0.60). Using the most recent classification of Papapanou et al., the majority of the study population was classified into stage 4 grade C (*n* = 90). This reclassification aligns more closely with the study’s results, lending greater coherence to the findings within the context of the new classification (Figure 6).

## 4. Discussion

Some patients are not always able to afford a fixed/implant-supported treatment. But a partial denture is not the most effective solution for periodontally affected patients. Avoiding a provisional removable partial denture can be beneficial for the periodontal treatment outcome. Another reason is obtaining additional time for patients with a shortened life expectancy (e.g., fatal disease). This retrospective study evaluated the survival and effective survival rate of mandibular incisors exhibiting >50% bone loss within a structured recall program.

This demonstrated a high long-term survival and effective survival rate. The re-evaluation at LFV confirmed that 79.6% of the patients retained all four incisors (survival rate) and an effective survival rate of 89.2%. This suggests that with adequate treatment and follow-up, the need for extraction of mandibular incisors with >50% bone loss at baseline can be significantly delayed or avoided. Extracted teeth repositioned with a dental splint could prolong the survival of the other incisors. All repositioned incisors survived until the end of the study follow-up, maintaining the functional and aesthetic integrity. Regarding the TF group (7.5%), the first failure occurred only after 7.6 years. Of this group, 71% had a failure range between 17.5 and 24.8 years. Two patients were classified as such because of an aesthetic replacement, but these patients still kept their proper teeth for a significant amount of time. After 20 years of follow-up, the survival probability is 78%, while the effective survival probability increased to 89%. With a challenging periodontal context (stage 4 grade C), long-term treatment is a viable option. Even the two patients with a maximum survival time of 32 years kept all incisors during the entire study.

The concept of “effective survival” includes repositioned and splinted mandibular incisors after extraction. Splinting does not restore lost periodontal support but rather modifies the biomechanical environment of periodontally compromised teeth [12]. By reducing individual tooth mobility, it redistributes the occlusal load across multiple teeth. The remaining periodontal ligament may undergo decreased mechanical stress, thereby reducing secondary trauma and creating conditions more favourable for functional stabilization. From a biological perspective, reduced mobility could facilitate periodontal ligament adaptation [20]. This does not imply periodontal regeneration but rather a functional adaptation of the reduced periodontium. During supportive periodontal therapy splinting may support long-term maintenance by allowing compromised teeth to function within a stabilized biomechanical unit. Consequently, the concept of effective survival should be interpreted primarily as functional maintenance rather than pure biological survival. Splinted teeth remained functional, contributing to the aesthetic aspect as well as the patient’s comfort. Nevertheless, their long-term prognosis depends on continued maintenance and inflammation control. This distinction between biological stability and functional preservation is important when interpreting long-term outcomes.

In the literature, only one study by Sonnenschein et al. (2022) [21] is relatively comparable. But only ‘at least one incisor with ≥50% breakdown’ was required; all patients received a splint; and at the baseline, they had 39 patients. Their 15-year follow-up is based on 4 patients (10.3%) of the original population. In this study at the same time point 65.5% (*n* = 61) were still present. The general periodontal health of their patient population was not evaluated.

The generally accepted risk factors (smoking, diabetes, etc.) did not show any statistically significance in the present research. This finding is likely attributable to the relatively small number of patients presenting with these risk factors, as the study was underpowered to draw definitive conclusions regarding their potential influence.

The importance of patient motivation and compliance is well documented [22,23]. The present study confirmed that good patient compliance almost doubled the effective survival and consequently the survival of the mandibular incisors in comparison with poor compliance. Tooth survival and patient compliance are likely associated underlying biological mechanisms. Regular supportive periodontal therapy repeatedly disrupts dysbiotic biofilm accumulation, thereby limiting reactivation of the host inflammatory response. The shift from CPITN 3 or 4 towards healthier categories observed at re-evaluation supports the concept that long-term inflammation control leads to periodontal tissue stability, rather than continued destruction. Consequently, maintenance therapy is both a behavioural factor, as well as a biologically driven intervention maintaining host–microbial homeostasis. Long-term effective survival of periodontal treatment together with patient education and regular maintenance leads to long-term survival of teeth. This was not only detectable for mandibular incisors but for the entire dentition. However, direct comparisons between studies remain limited, as differences in maintenance protocols, case selection, baseline disease severity and definition of tooth survival may substantially influence reported outcomes, as highlighted in recent systematic reviews and meta-analyses [24,25,26]. A comparison of the current results with the existing literature is difficult as the latter report solely on all teeth being splinted before initial periodontal therapy [27,28,29]. The current study primarily focusses on periodontal treatment of severely compromised teeth, with tooth splinting only in a limited number of cases (9.7%). Regarding the general periodontal health of the patient population, at GR 34 patients scored a CPITN of 0–2. When evaluating this 0–2 score at LV, there were 42 patients in this group, showing a shift from CPITN 3 towards the 0–2 group. A borderline statistical significance was observed for follow-up frequency as compared to survival and should be considered a clinically relevant factor. When patients experienced a recurrence of the periodontal colonisation during maintenance, a new subgingival debridement session was scheduled. Retreatment showed maintenance of periodontal stability, highlighting the effectiveness of only non-surgical retreatment over decades, as none of the patients underwent surgical treatment during the entire study period.

This study compared the periodontal classifications of Armitage and Papapanou et al., revealing key differences in clinical applicability. Under Armitage’s classification, no statistical difference in survival was found between chronic or aggressive periodontitis. Applying the Papapanou classification, the majority (97%) of subjects fell into the Stage IV, Grade C category. This homogeneity provided a more coherent representation of the clinical severity and aligns better with observed outcomes. The newer classification offers improved clinical relevance and reflects the progressive nature of the disease more accurately in the context of long-term follow-up.

van Winkelhoff et al. (1992) [30] introduced the adjunctive use of a combination antibiotic therapy in periodontal treatment. Their main target group were patients with an aggressive periodontitis characterized by the presence of Aggregatibacter Actinomycetemcomitans and Porphyromonas Gingivalis. Even with the available literature, no antibiotics were prescribed during the entire treatment, 1990–2022 The recent literature indicates that systemic antimicrobials are no longer a part of conventional first-line therapy. They are an adjunctive therapy, considered in specific periodontitis cases [31]. Due to the relevant negative consequences of systemic antimicrobials, their use should be restricted, being always prudent and rational and following protocols [32,33]. Although no antimicrobials were prescribed the effective survival rate is 89% up to 20 years. In selected patients, mechanical biofilm disruption and long-term inflammation control may be sufficient. This may be supported by the favourable outcomes achieved without systemic antimicrobials in this study.

The number of occluding units did not SS influence mandibular incisor survival, suggesting that functional loading alone is not a critical determinant. A. Käyser (1981) [34] introduced the shortened dental arch concept, confirmed multiple times [35,36], which is in line with the present results. Total tooth loss over the follow-up period was SS associated with mandibular incisor survival (*p* < 0.001). This highlights the broader systemic or behavioural implications of ongoing tooth loss, potentially reflecting patient-level factors such as compliance, general periodontal breakdown or reduced overall prognosis. Interestingly, biomechanical loading (e.g., bite force, occlusal trauma, etc.) alone might not be a primary risk factor of tooth failure when inflammatory control is achieved. This conclusion could be based on the absence of a significant effect of occluding units on survival [37]. The favourable long-term tooth retention observed in this cohort may be attributed to sustained control of periodontal inflammation following active therapy and supportive care. Resolution of inflammation reduces destructive host responses and limits further bone resorption, thereby allowing remodelling processes to establish a functionally stable, though structurally reduced, periodontium. In this context, long-term retention appears to reflect biological stabilization rather than complete regeneration [38].

## 5. Conclusions

The present study indicates that long-term preservation of severely periodontally compromised mandibular incisors is feasible when treatment is followed by structured supportive periodontal care. Patient compliance and sustained inflammation control appear to be decisive factors in maintaining stability over time. After 20 years the survival probability was 78%, and the effective survival probability, 89%.

These findings support a conservative, tooth-preserving strategy in cases with advanced bone loss, as extraction and prosthetic replacement may often be postponed or avoided. Functional and aesthetic integrity can be maintained for extended periods, provided that patients adhere to lifelong periodontal maintenance.

## Figures and Tables

**Table 1:** Reliability of measurements.

	Reliability Between the 2 Measurements			Reliability Between the 2 Consecutive Days		
	Day 1	Day 2	Day 1&2	1st measurement	2nd measurement	Mean
	ICC intraclass correlation			ICC intraclass correlation		
Perio probe	0.7730	0.7727	0.7729	0.9110	0.9437	0.9501
Calliper	0.7998	0.7888	0.7946	0.8661	0.9467	0.9407
e-Calliper	0.7646	0.8184	0.7898	0.8821	0.9718	0.9634
	Pearson correlation			Pearson correlation		
Perio probe	0.7730	0.7727	0.7698	0.9110	0.9437	0.9501
Calliper	0.7998	0.7888	0.7842	0.8661	0.9467	0.9407
e-Calliper	0.7646	0.8184	0.7890	0.8821	0.9718	0.9634

## Data Availability

The raw data supporting the conclusions of this article will be made available by the authors on request.
